# LFA-1 and ICAM-1 expression induced during melanoma-endothelial cell co-culture favors the transendothelial migration of melanoma cell lines in vitro

**DOI:** 10.1186/1471-2407-12-455

**Published:** 2012-10-05

**Authors:** Stephanie Ghislin, Dorian Obino, Sandrine Middendorp, Nicole Boggetto, Catherine Alcaide-Loridan, Frederique Deshayes

**Affiliations:** 1 Team « Regulation des Reponses Immunitaires ». Institut Jacques Monod, CNRS, UMR 7592, Univ Paris Diderot, Sorbonne Paris Cité, Paris, F-75205, France; 2 Imagoseine, plate-forme de cytometrie en flux, Institut Jacques Monod CNRS-Universite Paris Diderot, Paris, France; 3 Equipe : « Regulation des Reponses Immunitaires», INSTITUT JACQUES MONOD, CNRS-Université Paris Diderot, Bâtiment Buffon. 15 rue Hélène Brion, Paris, 75205 PARIS CEDEX 13, France

**Keywords:** Melanoma, Transendothelial migration, Metastasis, LFA-1, ICAM-1, HUVEC

## Abstract

**Background:**

Patients with metastatic melanoma have a poor median rate of survival. It is therefore necessary to increase our knowledge about melanoma cell dissemination which includes extravasation, where cancer cells cross the endothelial barrier. Extravasation is well understood during travelling of white blood cells, and involves integrins such as LFA-1 (composed of two chains, CD11a and CD18) expressed by T cells, while ICAM-1 is induced during inflammation by endothelial cells. Although melanoma cell lines cross endothelial cell barriers, they do not express LFA-1. We therefore hypothesized that melanoma-endothelial cell co-culture might induce the LFA-1/ICAM ligand/receptor couple during melanoma transmigration.

**Methods:**

A transwell approach has been used as well as blocking antibodies against CD11a, CD18 and ICAM-1. Data were analyzed with an epifluorescence microscope. Fluorescence intensity was quantified with the ImageJ software.

**Results:**

We show here that HUVEC-conditioned medium induce cell-surface expression of LFA-1 on melanoma cell lines. Similarly melanoma-conditioned medium activates ICAM-1 expression in endothelial cells. Accordingly blocking antibodies of ICAM-1, CD11a or CD18 strongly decrease melanoma transmigration. We therefore demonstrate that melanoma cells can cross endothelial monolayers in vitro due to the induction of ICAM-1 and LFA-1 occurring during the co-culture of melanoma and endothelial cells. Our data further suggest a role of LFA-1 and ICAM-1 in the formation of melanoma cell clumps enhancing tumor cell transmigration.

**Conclusion:**

Melanoma-endothelial cell co-culture induces LFA-1 and ICAM-1 expression, thereby favoring in vitro melanoma trans-migration.

## Background

Metastatic melanoma account for most skin cancer deaths. When diagnosed early, primary, non-disseminated, tumors are successfully eliminated through excision. However, in about 20% of the cases, dissemination of tumor cells leads to aggressive forms of cancers highly refractory to chemotherapy, with a median survival rate of 6 months [1]. Uncovering molecules required for melanoma metastasis is therefore essential.

Hematogenous metastasis of cancer consists of several steps enabling cancer cells to intravasate, to survive in the blood circulation and to adhere to the vessels, eventually extravasating and establishing new metastatic lesions. Extravasation of most cancer cells largely mimics leukocyte transendothelial migration from the blood flow into sites of tissue inflammation [2]. This controlled process involves the multistep action of traffic signals and adhesion molecules that mediate rolling, adhesion and transendothelial migration of lymphocytes [3].

The role of cell adhesion molecules (CAMs), such as intercellular cell adhesion molecule-1 (ICAM-1), vascular endothelial cell adhesion molecule-1 (VCAM-1), E-selectin, and P-selectin, has been studied extensively in the process of inflammation [4]. Indeed, leukocyte adhesion during inflammation is thought to proceed in a cascade-like fashion, in which selectins are responsible for leukocyte capture and rolling, and integrins for mediating firm adhesion and transmigration [5,6]. Among these integrins, Leukocyte Function-Associated antigen-1 (LFA-1; α_L_β_2_) composed of two chains, CD11a and CD18, has been extensively described for its essential role in leukocyte extravasation [2,7]. It functions as a receptor for ICAM-1 (CD54) [8-10]. Besides its role in the firm adhesion of leukocytes to the endothelium, it appears dominant in transendothelial migration [7,11]. In addition numerous studies have shown that complete inhibition of CD18, or genetic mutations in CD18 profoundly reduce leukocyte transmigration at sites of inflammation [12].

Junctional Adhesion Molecules A (JAM-A) can also interact with LFA-1 via its second membrane-proximal Ig domain [13,14]. During leukocyte transendothelial migration, the homophilic transendothelial interactions between two molecules of JAM-A must be disrupted to enable a migrating leukocyte to pass through junctions [15] and it has been evidenced that LFA-1 binding to JAM-A destabilizes the JAM-A homophilic interaction, thus allowing transendothelial migration to proceed [16].

LFA-1 has been studied in different tumors, for instance myelomas and gastrointestinal carcinomas. It has been shown that expression of LFA-1 correlates with the aggressiveness of myeloma [17] and is present in metastatic gastrointestinal carcinomas [18].

In melanoma cell lines, LFA-1 cell-surface expression is not detected. Towards a molecular explanation to the high capacity of melanoma tumor cells to metastase, two groups proposed that melanoma cells interact with neutrophils, thereby suggesting that neutrophils might be used as carriers by the tumor cells [19-21]. Liang *et al.*[19] have notably demonstrated that under IL-8 signaling, melanoma interact with polymorphonuclear neutrophils (PMNs) through the binding between ICAM-1 on melanoma cells and β_2_ integrins on PMNs. The authors also showed that this interaction facilitates melanoma cell adhesion to the endothelial cells and subsequent extravasation by a shear-rate dependent mechanism [21].

However during our studies of melanoma metastasis, we observed that melanoma cell lines have the capacity to transmigrate through endothelial monolayers in the absence of PMNs. We therefore hypothesized that melanoma-endothelial cell co-culture might induce the ICAM-1/LFA-1 ligand-receptor interaction. In this manuscript we studied three human melanoma cell lines with differential transmigration capacities. We provide evidence that melanoma supernatants induce ICAM-1 expression on HUVEC cells, and that LFA-1 can be detected on melanoma cell lines when using HUVEC-conditioned medium. Further confirmation was obtained through the use of either ICAM-1 or LFA-1 blocking antibodies introduced during the co-culture and show that they strongly impair melanoma transmigration.

## Material and methods

### Cell lines and cell culture

The melanoma cell line SLM8 kindly provided by M. Viguier (Service de dermatologie, Hopital Saint-Louis, Paris, France), is derived from a lymph node metastasis. The 1205LU cell line, a generous gift by A. Mauviel (Institut Curie/CNRS UMR 3347/Inserm U1021), is derived from the lung metastatic WM793 cell line. The A375 cell line was purchased from the ATCC. HUVEC cells were kindly provided by C. Nahmias (Institut Cochin, Paris, France). A375, 1205LU and SLM8 human cell lines were grown in DMEM/F12 (Invitrogen, Cergy-Pontoise, France) supplemented with antibiotics and 10% fetal calf serum (FCS) in a 5% CO2 atmosphere (hereafter named FCS complete medium). HUVEC cells have been cultured in high glucose DMEM containing glutamax, 1% antibiotics and 10% FCS in a 5% CO2 atmosphere.

### Conditioned medium

To obtain conditioned medium cells were grown to confluence during 48hrs before collecting the supernatant. The medium was diluted to 3/4 with FCS complete medium (1/4) then added for 24hrs to the target cells plated at 80% of confluence. The basal negative control was obtained with only FCS complete medium.

### RNA extraction and semi-quantitative PCR

Total RNA was extracted using the RNeasy kit (Qiagen, Courtaboeuf, France) following the manufacturer’s instructions. cDNA was synthesized from 2 μg of total RNA using 400 ng oligo(dT) (Invitrogen, Cergy-Pontoise, France), 20 units RNAsin RNase inhibitor (Promega, Madison, Wisconsin, USA) and 8 units Omniscript reverse transcriptase following the manufacturer’s instruction. PCR was performed by an initial denaturation step at 95°C for 5 min, followed by 30 cycles at 95°C for 45s, 60°C for 45s (GAPDH) and 72°C for 1min. Primer sequences for human GAPDH were: 5^′^–GTCGTATTGGGCGCCTGGTCAC–3^′^ and 5^′^–AGGGGCCATCCACAGTCTTCTG–3^′^. All primer sequences, hybridization temperature, number of cycles and PCR conditions for other genes tested are summarized in Table 1.

**Table 1:** Primer sequences and PCR settings

**Gene name**	**Primer direction**	**Sequences (5’-3’)**	**Number of cycle**	**Hybridization temperature**
CD11a	Forward	GGGAATGACCTTGGCAACAGACCCCACAGAT	34	58
	Reverse	GGGTCTCCTGACTCTCCTTGGTCT		
CD18	Forward	CATCAGAGCTGCTGTAGAGC	31	60
	Reverse	GCTGACCTTGAACTTCGTGC		
ICAM-1	Forward	TGCAGCACCTCCTGTGACCA	25	60
	Reverse	CAGTTCCACCCGTTCTGGAG		
E-Selectin	Forward	GGACTGCGTGGAGATCTACA	30	61
	Reverse	AGCCAGGGTCACACTTGCAA		
N-Cadherin	Forward	GAAGGATGTGCATGAAGGAC	30	56
	Reverse	AGTTAAGGTTGGCTTCAGGC		
GAPDH	Forward	GTCGTATTGGGCGCCTGGTCAC	15	60
	Reverse	AGGGGCCATCCACAGTCTTCTG		

### Flow cytometry

Cells (5 × 10^5^) treated with conditioned medium from a 48hrs of HUVEC cells culture were harvested in cold phosphate-buffered saline (PBS) containing 1 mM ethylenediaminetetraacetic acid (EDTA) and incubated for 20 min with 2% fetal calf serum in PBS. Specific direct primary antibodies CD11a antibody (FAB35951A) and CD18 (FAB1730P) from R&D system (Minneapolis, MN, USA) or isotypic control antibody (BD Pharmingen, San Diego, CA, USA) were used at 1 μg ml. After three washes, cell death was monitored by propidium iodide (5 μg ml) uptake and fluorescence was analyzed in a cytometer from DAKO (Trappes, France).

### Trans-endothelial migration of melanoma

Trans-endothelial migration of melanoma was performed as previously described [22]. Briefly, 5 × 10^4^ HUVEC cells were seeded on 2mg/ml type I collagen-coated Transwell culture inserts with 8 μm pores (Greiner Bio-One SAS, Courtaboeuf, France) and grown for 2 days. Melanoma cells were labeled with 10 μM of the lipophilic fluorescent dye DiO (Molecular Probes, Invitrogen, Cergy-Pontoise, France) for 20 minutes at 37°C. 5×10^4^ fluorescent melanoma cells were added to wells containing either 1μg/ml of isotypic control (IgG from BD Pharmingen, San Diego, CA, USA); or anti-CD11a (ab3981; Abcam; Paris, France), anti-CD18 (ab8220; Abcam; Paris, France) or anti-ICAM-1 (MAB2146Z; Millipore; Molsheim France) antibodies in the upper chamber. A chemotactic gradient was created by addition of 10% FCS to the lower chamber. Melanoma cells were allowed to migrate at 37°C and 5% CO2 for 48 hours.

To remove non-migrating cells, the ones on the upper face of the filter were gently scraped using a cotton swab. Cells on the lower face were washed in PBS, fixed with 4% formaldehyde for 10 min and washed in PBS. Nuclei were then labeled with 1μg/ml DAPI for 5min and cells were washed again. The migrating melanoma cells were observed under an epifluorescence microscope using a magnification of x100. Three to five pictures of adjacent fields of the central zone of each Transwell were taken. Fluorescence intensity was quantified with the ImageJ software (US National Institutes of Health, Bethesda, MD, USA). Histograms display the data obtained with 3 independent experiments, and all the experiments were performed in duplicate. The p value has been calculated by an ANOVA statistical test.

### Human cytokine array

The detection of cytokines’ production by the three melanoma cell lines has been tested after 24hrs of culture in 12ml of culture medium using the Proteome Profiler™ Array Human Cytokine Array Panel A according to the manufacturer’s instructions (ARY005; R&D systems Minneapolis, USA). Hence, melanoma cells have been tested on nitrocellulose membranes each containing 36 different anti-cytokine antibodies spotted in duplicate. Experiments have been done in duplicate.

## Results

### Melanoma cells transmigrate through endothelial cells

Transwell experiments mimicking transendothelial migration were performed with three melanoma cell lines. Briefly, HUVEC cells (Human Umbilical Vein Endothelial Cells) are grown as a monolayer to confluence on a type I collagen matrix layer poured onto a microporous membrane. Melanoma cells, stained with a lipophilic fluorescent dye, are then allowed to cross this barrier for 48hrs. The corresponding membranes of the Transwells are photographed prior to the quantification of fluorescent cancer cells, located at the lower part of the membrane.

**Figure 1:**
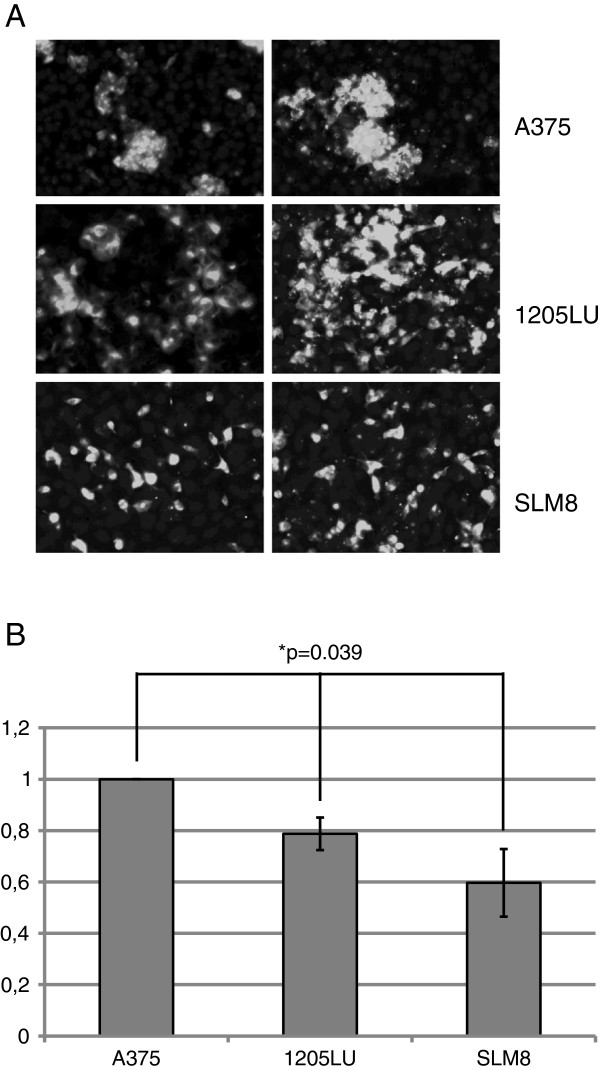
***Differential transendothelial migrations of A375, 1205LU and SLM8 cell lines.*A** HUVEC cells were seeded on type I collagen-coated Transwell culture inserts. DiO-labelled melanoma cells were added to the upper chamber and allowed to migrate for 48 h. Cells on the lower face of the inserts were fixed and labeled with DAPI prior to their observation under an epifluorescence microscope. **B** The histograms represent the quantification of the data obtained from 3 independent experiments, and all the experiments were performed in duplicate. The A375 cell line was considered here as having a 100% transmigration efficiency.

As seen in Figure 1, all three cell lines have the capacity to transmigrate. The A375 cell line presents the highest efficiency of transmigration while the SLM8 cell line displays the weakest capacity of transendothelial migration. The 1205LU cells have an intermediate phenotype. Interestingly the efficiency of transmigration of these cell lines appeared to correlate with the formation of clumps (Figure 1A). Indeed A375 cells tend to aggregate as clumps whereas the SLM8 cells transmigrate mostly as single cells. 1205 LU cells displayed an intermediate phenotype with a mixture of small clumps and single cells. In order to determine the importance of passive passage of melanoma cell lines, we performed transwell experiments as described below without HUVEC cell monolayer and without chemotactic gradient. In these conditions, the ability of melanoma cells to pass through the microporous membrane was almost inexistent and no significant difference was observed between the three cell lines (p value >0.05) (data not shown).

These data indicate that melanoma cells have the capacity in vitro to cross endothelial cell monolayer, although PMNs are not present. We therefore studied the molecular mechanisms leading to this phenomenon.

### LFA-1 is involved in melanoma trans-endothelial migration in vitro

**Figure 2:**
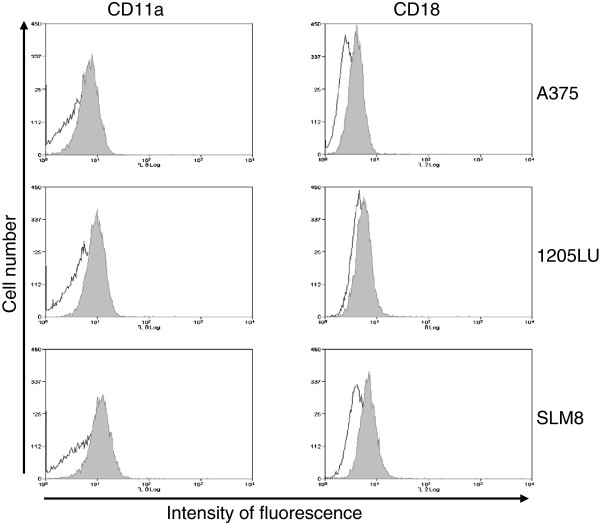
***Expression of CD11a and CD18 in melanoma A375, 1205LU and SLM8 cell lines with conditioned medium.*** Cell-surface expression of CD11a and CD18 on indicated melanoma cell lines treated for 24 hrs with HUVEC conditioned medium was analyzed by flow cytometry. Isotypic controls are represented as empty histograms and specific antibody-labelling is displayed as shaded histograms. Histograms obtained with cells incubated with FCS-complete medium and labeled with specific antibodies, which overlap with the isotypic control are not shown. Data from obtained with 3 independent experiments.

None of the three melanoma cell line expresses the LFA-1 subunits, CD11a or CD18 in classical culture condition (data not shown). However, when cultured in the presence of HUVEC supernatant for 24 hours, all three cell lines display a weak but reproducible expression of both chains at their cell surface (Figure 2).

**Figure 3:**
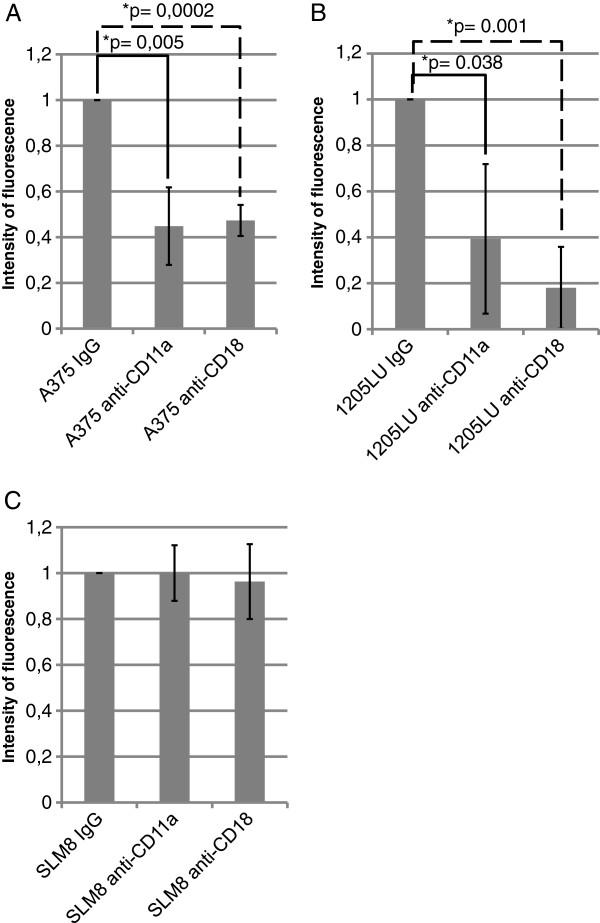
***Effect of CD11a and CD18-blocking antibodies on the transendothelial migration of A375, 1205LU and SLM8 cell lines.*** The experiments were performed as detailed in Figure 1, except that 2μg/ml of CD11a or CD18-blocking antibodies were introduced in the upper chamber of the Transwells when indicated. Histograms represent 3 independent experiments. In each experiment each condition was analyzed in duplicate.

In order to value the importance of LFA-1 in melanoma transmigration, blocking antibodies specifically directed against CD11a or CD18 were introduced during the transmigration assays. An anti-IgG antibody was used as a negative control. Concerning the two cell lines which transmigrate the most efficiently, A375 and 1205LU, a decrease of their trans-endothelial migration was observed when either CD11a or CD18 blocking antibodies were present (Figure 3AB). With the SLM8 cell lines, neither CD18 nor CD11a blocking antibodies affect the transmigration efficiency (Figure 3C).

### Melanoma cell lines enhance the expression of ICAM-1 on the HUVEC cells

**Figure 4:**
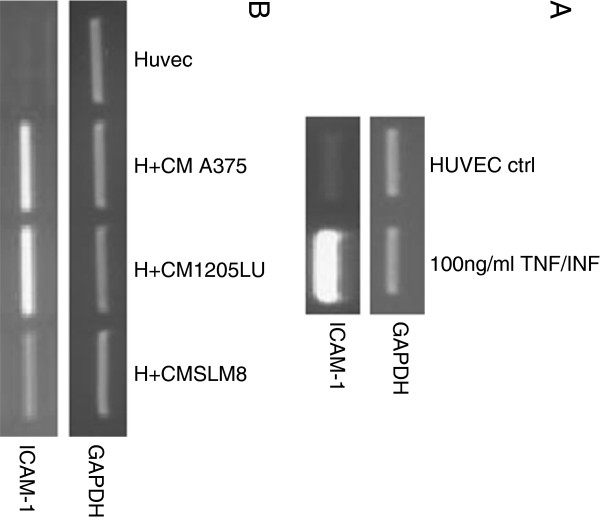
***Conditioned mediums from melanoma cell lines enhance transcript expression of ICAM-1 in HUVEC cells.*** Semi-quantitative PCRs were performed to detect expression of ICAM-1 transcripts. **A** HUVEC cells were treated either with TNF-α and IFN-γ at 100ng/ml or **B** with conditioned medium from A375 (H+A375), SLM8 (H+SLM8) and 1205LU (H+1205LU) after 48hrs of cell culture. GAPDH is used as a DNA amount control. Data were obtained from 3 independent experiments.

In normal conditions, HUVEC are known to lack high expression of ICAM-1. However, it has been shown that this level is strongly increased under inflammation [9], as exemplified on Figure 4A, where ICAM-1 transcript expression is highly induced in HUVEC cells treated with 100ng/ml of TNF-α and IFN-γ (Figure 4A). 

As exogenous inflammation molecules are not used in the transmigration assays displayed in this report, we wondered if melanoma cell lines could induce ICAM-1 expression in HUVEC cells. To answer this question, conditioned medium was prepared from the melanoma cell lines after 48hrs of culture. The HUVEC cell line was next cultured with this conditioned medium and ICAM-1 transcript expression was analyzed. Figure 4B shows that ICAM-1 is up-regulated by the conditioned medium originating from all three melanoma cell lines, more efficiently with the A375 and 1205LU cell supernatants.

**Figure 5:**
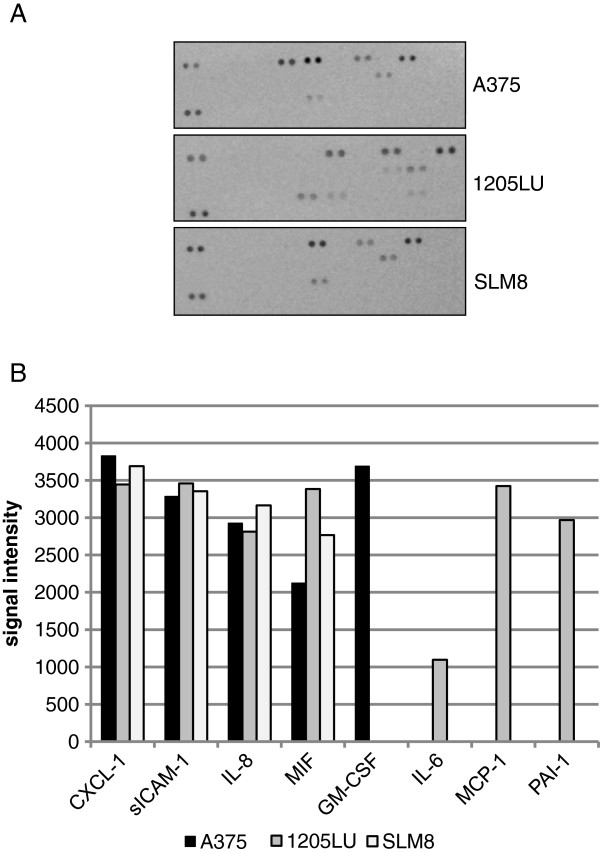
***Melanoma cell lines display different cytokine expression profiles.*A** Melanoma cells were cultured for 24hrs in 12ml of DMEM added with 10% FCS. The supernatant was next tested for the presence of 36 different cytokines. **B** The histograms represent the quantification by Image J software of the spots obtained on the nitrocellulose membranes. Data were obtained from 2 independent experiments.

Interestingly, when analyzing the effect of the conditioned medium from melanoma cell lines on different relevant genes, we observed that IL-8 and VEGF were also induced in HUVEC cells (data not shown). However the profile of gene expression was slightly different from the one obtained with IFN-γ and TNF-α. To identify the cytokines produced by melanoma cells, a cytokine array was next performed (Figure 5). Hence we noticed that melanoma cells, mainly the A375 and 1205LU cell lines, which have the higher capacities of transmigration, secrete pro-inflammatory cytokines, namely GM-CSF and IL-6, which have been described to up-regulate the expression of ICAM-1 [23,24]. In addition all three cell lines secrete molecules such as IL-8 and CXCL-1 (Figure 5). We demonstrated that cells positive for surface expression of ICAM-1 (data not shown) also secrete sICAM-1, which has been extensively published as being up regulated in many tumors [25], notably in melanoma where it has been shown to be associated with disease progression [26,27] and proposed as a prognosis marker [28,29]. Of interest, and to corroborate with our hypothesis, cell lines expressing high amounts of GM-CSF were displaying metastatic competence and invasion [30,31]. Indeed the A375 cell line, secreting GM-CSF, has the highest rate of transmigration. Alternatively, one might propose that PAI-1 might be of interest for the 1205LU cell line transmigration, as PAI-1 was reported to affect the degradation of the extracellular matrix [32,33]. 

**Figure 6:**
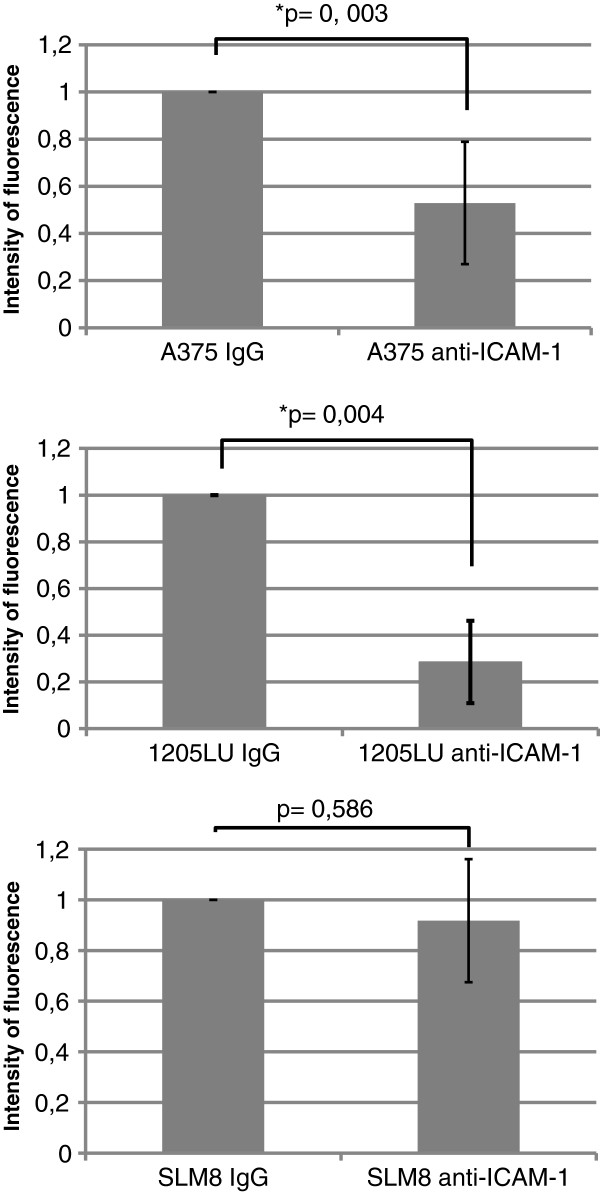
***Effect of ICAM-1-blocking antibodies on the transendothelial migration of A375, 1205LU and SLM8 cell lines.*** The experiments were performed as detailed in Figure 1, except that 2μg/ml of ICAM-1-blocking antibodies were introduced in the upper chamber of the Transwell when indicated. Histograms represent 3 independent experiments. In each experiment each condition was analyzed in duplicate.

Having shown that melanoma cells can induce ICAM-1 expression on HUVEC cells, it was necessary to provide evidence of ICAM-1 implication in the transmigration. ICAM-1 blocking antibodies were assayed on the same cell lines. As seen on Figure 6 blocking ICAM-1 impairs transmigration of A375 and 1205LU, but not that of SLM8, which is consistent with our previous observations of a poor expression of ICAM-1 on endothelial cells when incubated with SLM8-conditioned medium.

We therefore conclude that preferentially the A375 and 1205LU melanoma cell lines can transmigrate through the endothelial cells due to a binding between ICAM-1 expressed by the HUVEC cells and LFA-1expressed by tumor cells during their co-culture.

### LFA-1 is involved in clumps’ formation

**Figure 7:**
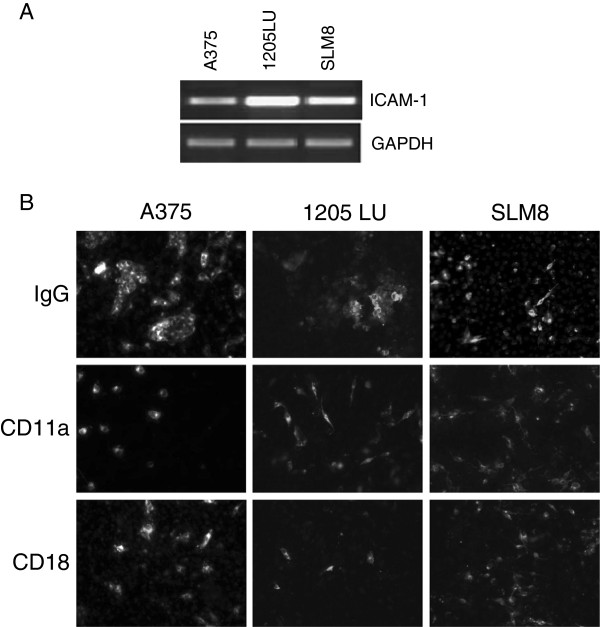
***Effect of CD11 and CD18-blocking antibodies on the formation of clumps.*A** Semi-quantitative PCRs were performed to detect the expression of the ICAM-1 transcript. GAPDH is used as a DNA amount control. **B***A375, 1205LU and SLM8 cell lines* were treated with 2 μg/ml of CD11a or CD18-blocking antibodies as indicated. Melanoma cells were labeled with DiO then fixed and labeled with DAPI prior to their observation under an epifluorescence microscope using a magnification of x10. Data were obtained from 3 independent experiments.

As ICAM-1 expression is often detected on melanoma cell lines, we hypothesized that clumps might assemble due to an interaction of LFA-1 and ICAM-1, and that this cell association might enhance melanoma transmigration capacities as described in some tumors [34]. All three melanoma cell lines express ICAM-1 and LFA-1 in the presence of conditioned medium from HUVEC cells (Figure 2). However only A375 and 1205LU form cell clumps (Figure 7), suggesting that these sole cell lines possess functional partners for the interaction. To further confirm the role of LFA-1 in the generation of cell clumps, we examined the persistence of clumps by using CD11a and CD18 blocking antibodies. As shown in Figure 7, clump formation is no longer observed when A375 and 1205LU cell lines were treated with anti CD11a and anti CD18. These treatments do not affect the morphology of SLM8 cells. Consequently we propose that trans-endothelial migration of melanoma would be a cooperating endeavor, with clumps favoring the binding to endothelial cells via the presence of LFA-1 on melanoma cells. 

## Discussion

The recruitment of lymphocytes to sites of inflammation involves a sequence of rolling along capillary vessel walls, followed by chemokine induced arrest and migration across a tight layer of vascular endothelial cells [35,36]. Invasion of tumor cells to secondary sites is often compared with lymphocyte transendothelial migration since several common cell adhesion molecules, such as selectins and integrins, are involved in both lymphocyte and tumor cell migration. It has previously been shown that following secretion of IL-8 in the tumor microenvironment, the PMN facilitated melanoma extravasation via the binding of *β*2 integrins on PMNs and ICAM-1 on melanoma cells [37]. In a congress report, it was mentioned (although not confirmed through a publication) that the co-culture of HUVEC cells and a melanoma cell line from a lymph node metastasis promotes the expression of αVβ3 facilitating melanoma extravasation [38]. In this report, we focused on the direct role of a major integrin, LFA-1 and its counter receptor ICAM-1 in melanoma transmigration. To investigate this hypothesis we used three melanoma cell lines displaying different efficiencies of transendothelial migration but similar expression of the tested integrin. With blocking antibodies against these integrins and ICAM-1, we showed that LFA-1 promotes melanoma cells transmigration in vitro. In addition we provide evidence that LFA-1 seems to be responsible for clumps’ formation, which could enhance further their extravasation capacities. This is consistent with previous reports examining the migration of dendritic cells [39] or extravasation of some tumors [40,41].

**Figure 8:**
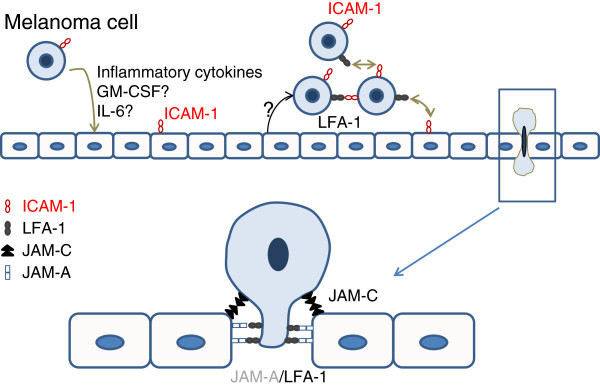
***Model of melanoma transmigration.*** According to these results and to those of our previous publication [22] a model of melanoma transmigration can be proposed involving integrin and JAM proteins

The expression on melanoma cell lines of such molecules which are normally expressed on immune cells might appear unexpected. However other immune cell-specific molecules have already been shown to be expressed on these tumors. Indeed, we have previously demonstrated that MHCII (major histocompatibility complex, Class *II*), in contrast with melanocytes, is constitutively expressed on melanoma cells. We have reported that the deregulation of HLA-D genes is due to the abnormal constitutive expression of the lymphocyte-specific isoform of class II transactivator (B-CIITA) [42]. This expression is driven by the MAPK cascade [43] and associated with tumour progression and metastatic dissemination [44]. MHCII is not the only immune protein expressed by melanoma cells. Indeed, as exposed above, LFA-1 is able to bind to JAM-A. In a previous publication [22] we provided evidence of the differential roles of JAM proteins, usually expressed by lymphocytes, in melanoma transendothelial migration, where JAM-C promotes their transendothelial migration, while expression of JAM-A inhibits this phenomenon. Although both JAMs are described as adhesion molecules, JAM-A is involved in the tight junctions’ maintenance of endothelial cells [45] and it has been reported that LFA-1 binding to JAM-A destabilizes the JAM-A homophilic interaction, thus disrupting this connection, and facilitating its transendothelial migration [16]. Since HUVEC cells express JAM-A (data not shown) this phenomenon could also be, in part, responsible for the transmigration we observed, in conjunction with the interaction between LFA-1 of melanoma cells and ICAM-1 of endothelium and with the formation of melanoma cell clumps (Figure 8), Alternatively an interaction between Mac-1 and ICAM-1 might be relevant, as anti-CD11b antibodies can reduce SLM8 transmigration, while it does not alter the migration of the two other cell lines (data not shown). 

Patients with metastatic melanoma have a reduced life span in the range of 6 months. Limited therapeutic treatments explain this poor prognosis. Our findings showing that CD18 blockade impairs melanoma cell transmigration might provide a new tool to control melanoma metastatic mechanism.

## Competing of interest

The authors’ declare that they have no competing interests.

## Authors’ contributions

SG, DO, CAL and FD designed the study. NG carried out the cytometry analysis. SG, DO, SM and FD performed the data analysis and interpretation. SG, CAL and FD wrote the manuscript. All authors read and approved the final manuscript.

## Pre-publication history

The pre-publication history for this paper can be accessed here:

http://www.biomedcentral.com/1471-2407/12/455/prepub
